# The inner fire: the shifting paradigm of complement system in aging

**DOI:** 10.3389/fimmu.2026.1795833

**Published:** 2026-04-14

**Authors:** Paola Triggianese, David Della-Morte

**Affiliations:** 1 Department of Biomedicine and Prevention, University of Rome Tor Vergata, Rome, Italy; 2 Immunolgy and Allergology, HAE Reference Center, Tor Vergata University Hospital, Rome, Italy; 3 Nurition and Geriatric Division, Tor Vergata University Hospital, Rome, Italy; 4 Department of Neurology, Evelyn F. McKnight Brain Institute, Miller School of Medicine, University of Miami, Miami, FL, United States

**Keywords:** aging, autoimmunity, complement system, complosome, immunosenescence, metabolism, neuroinflammation

## Abstract

The complement system (CS) is a key component of innate immune system that could be activated through pathways converging in activating C3 to cell-specific targeting. Besides its first-line defense against infection, CS serves as a bridge to adaptive immunity by modulating T-cell function and enhancing B-cell-mediated responses, thereby strongly contributing to the overall immune homeostasis. While deficiencies of CS components often result in increased susceptibility to infection, dysregulation of CS activation is associated with autoimmunity and chronic inflammation. Given the ability of the CS to rapidly respond to pathogen-associated molecular patterns along with its redundancy, it also relies on strictly regulated checkpoints to prevent unintended host damage. During aging, the CS undergoes a relevant shift: in elderly, the persistent low-grade inflammation leads to the continuous activation of CS that in turn contributes to the sustained chronic inflammation (“inflammaging”): CS thus might correlate with the increasingly well-known age-related degenerative diseases. However, during aging, CS might act like a two-faced Janus: on one hand, CS drives persistent chronic inflammation by acting as a mediator of inflammation; on the other, elevated levels of CS proteins may act as immunomodulatory agents and prevent disorders associated with CS abnormalities, such as neuroinflammation and autoimmune diseases. This review synthesizes the emerging evidence of potential protective role of the aging-related CS dysregulation in elderly. We explore how CS in elderly people modulates a sophisticated network in immunosenescence, neuroinflammation and autoimmunity. Understanding this “switch” of CS during aging will be essential for designing novel strategies for therapeutic modulation of CS-driven inflammation and damage while preserving its potential role in defense and repair.

## 1 Introduction

Complement system (CS) was discovered by chance and received its name for a supposed activity of *complementing* antibodies’ function (1). Nevertheless, CS is more than just an adjuvant to the adaptive immune system: it is the main mechanism of self-defense and a key bridge between innate and adaptive immunity (2). Historically known as composed of over 50 soluble and liver-derived proteins activated via the classical, lectin, and alternative pathways, CS is then better described as not confined to plasma but as a tissue-based system. Notably, CS can be activated locally, at the sites of action, to exert its main activities of host defense against foreign pathogens, independently of the adaptive immune system. However, the classical and lectin pathways are activated generally upon recognition of exogenous stimuli while the alternative pathway is constitutively active at low levels, and this refers to as the *tickover mechanism* allowing the CS to stay primed for rapid activation (3). Nevertheless, altered-self signatures on apoptotic or hypoxic cells can activate CS: all the CS activation pathways converge to the formation of C3 convertases, which cleave the abundant plasma protein C3 into an anaphylatoxin (i.e., C3a) which recruits immune cells and an opsonin fragment (i.e., C3b) which “tags” targets for phagocytosis thus inducing effector functions (4). Pathways converge upon a common final step: the formation of lytic membrane attack complex (MAC) directly causes the creation of pores in the pathogen cell’s lipid bilayer inducing subsequent lysis (4). Evidence has revealed that key CS components, particularly C3 and C5, are active not only in extracellular immunity but also within cells: the “complosome” is an intracellular CS which serves as a regulator of crucial cellular processes, including oxidative stress, metabolism, and cell survival (5, 6). Notably, complosome also participates in sterile inflammation - the immune response triggered by damage-associated molecular patterns (DAMPs) rather than pathogens: in this context, the interplay between the complosome and the NLRP3 represents a key orchestrator of basic cellular processes (7). Most complosome proteins are generated directly within the cells: however, they can also be further internalized from the cell surface membrane and extracellular sources (8). Two major factors of sterile inflammation are the activated complement cleavage fragments C3a, C5a, and C5b-C9 (MAC), and ATP released from stressed cells: they directly increase the level of reactive oxygen species (ROS) in the cellular cytosol by activating mitochondria through corresponding receptors. The subsequent release of ROS also leads to the activation of the NLRP3 inflammasome which is a key player in tissue homeostasis, by balancing sterile inflammation and supporting tissue repair (9). Evidence in hematopoietic stem cell (HSC) aging is available describing reduced HSC number and mechanisms of functional decline of these aged HSCs including mitochondrial stress, DNA damage, and SIRT2-NLRP3-caspase 1 axis (9). That abnormal NLRP3 activation has been not described in young, healthy HSCs (9). During aging, persistent low-grade inflammation promotes the continuous activation of CS that in turn contributes to sustained chronic inflammation (“inflammaging”). However, during aging, CS might have a dual role: on one hand, CS drives persistent chronic inflammation by acting as a mediator of inflammation; on the other, elevated levels of CS proteins may act as immunomodulatory agents and prevent disorders associated with CS abnormalities, such as neuroinflammation and autoimmune diseases. While evidence on links between complement biology and autoimmune-inflammatory diseases is established, in an aging context a combination of emerging evidence and evidence-based hypotheses warrants an increase in research efforts in this field.

### 1.1 CS and aging: the shifting paradigm

Aging is a sophisticated, irreversible, and multifactorial process characterized by the accumulation of molecular abnormalities that progressively impair cellular function (10). Indeed, aging is a dynamic and time-dependent process being characterized by the progressive and ever-increasing susceptibility to morbidity (11). As documented in the literature, relevant hallmarks of aging include genomic instability, epigenetic alterations, progressive telomere shortening, and impaired mitochondrial function, alongside cellular senescence and both intrinsic and extrinsic factors (12). The aging-associated immunologic alterations represent potential promising targets as well as outcome measures for therapeutic interventions in frail olders: intriguing research and clinical strategies are ongoing to advance comprehensive care for elderly patients (**Table 1**). As the intricate network of mechanisms underlying aging is still a difficult process to define, inflammation certainly represents a key feature associated with the aging process (13). In this context, CS acts as a key contributor to age-related degenerative disorders given its role in tissue injury and damage (14). In the age-related deterioration process, oxidative stress could serve as a primary driver, given the endogenous ROS-related cumulative damage (15, 16). Current evidence, not specific to the aging context, clearly describes that oxidative stress is a potent trigger of CS activation: in particular, ROS can lead to a non-enzymatic cleavage of C5 promoting MAC formation and, thus, cellular lysis. In addition, chronic oxidative stress leads to tissue alterations that in turn trigger chronic inflammation, while activating CS. Regulators of CS activation - both soluble (such as Factor H, Factor I, C4bp, clusterin) and at the cellular level (such as CR1, CD46/MCP, CD55/DAF, CD59) – physiologically block and/or inactivate CS-mediated inflammation thus preserving self-tolerance and tissue integrity - while ensuring host defense (17). As described, mitochondrial function declines with aging, and leads to enhanced ROS production: oxidative stress and inflammatory pathways have crucial impact on aging and, in this context, mitochondria act as a key player in the interplay between oxidative stress, inflammation, and aging (10). The CS activation under oxidative stress includes both antibody-independent (mainly through lectin pathway) and antibody-dependent - through antibodies binding to neo-epitope generated by oxidative damage - pathways. Furthermore, evidence from the general biological context rather than aging-specific data documents that CS activation leads to the formation of C3a and C5a that recruit neutrophils and macrophages, contributing to a persistent ROS production (18). Based on this general context (non age-specific), it could be reasonably hypothesized that vicious cycle - in which CS and oxidative stress amplify each other - results in progressive damage to aging tissues. The complement receptor type 1 (CR1, CD35) regulates the activity of complement by interfering with its ligands C3b, C4b, C3bi, C1q, MBL, and ficolins thus inhibiting the activation of complement. Evidence supports the role of CR1 on erythrocyte surface (CR1/E) as the main “cooling system” of CS activation and inflammation on endothelial cells. Authors analyzed the acquired decrease of the CR1/E from COVID-19 patients and attempted to provide age-stratified results (19). They reported that values for CR1/E density from controls were similar between elderly donors and donors under 65; however, in COVID-19 patients, during the course of the disease, CR1/E continued to decrease when compared to initial CR1/E determination and age resulted a major predictive factor of decreased CR1/E thus suggesting the over activation of handling and clearance of immune complex or complement fragments (19). Indeed, a larger proportion of CR1/E can be lost in acute situations such as infections, Alzheimer’s disease (AD), and autoimmune diseases (19–21).

**Table 1:** Current trials on interventions in older adults focusing on immune age (cellular biomarkers, cytokines, cell repertoire) and immune fitness (a dynamic balance between inflammation, Complement System, immune cells).

ID and status	Title	Study population	Design	Primary outcomes
NCT06513520 recruiting	SENIOR HLA DR	Hospitalized multi-pathological subjects over 75 years old in Geriatric Services	Observational Prospective Cohort	Expression of Monocyte HLA-DR
NCT06907329 recruiting	Nicotinamide Mononucleotide Sustained-release Tablets on Immunosenescence and Metabolism	Adults 50-70 years of age with Metabolic Disorders	Randomized Interventional Parallel Assignment	CD3+CD8+CD27-CD28- T cells as a percentage of CD3+CD8+ T cells
NCT04763291 Active, not recruiting	Cardiovascular and InflammAging Study: effects of long-term consumption of plant-based supplements	Adults 50-80 years of age	Randomized Interventional Parallel Assignment	Oxidative stress markers, cytokines. Secondary outcomes: neurotrophins, mitochondrial DNA
NCT03944603 Active, not recruiting	Longitudinal Innate Immunity and Aging Study (LIIA)	Adults 60-89 years of age without significant cognitive decline	Observational Prospective Cohort	Levels of immune protein markers and exosomal innate immune in the blood and in the spinal fluid
NCT05421325 recruiting	Assessment of QBKPN Site-Specific Immunomodulator efficacy in Improving Innate Immune Function	Adults 65 years of age or older residing in the community, in independent-living, assisted-living and long-term care	Randomized, double-blind, placebo-controlled	Secondary outcomes: innate immunity
NCT05857241 not yet recruiting	Therapeutic Fasting and Immune Aging (JÛVENILE)	Adults 65 years of age or older residing institutionalized in long term care units or establishments for the elderly	Single group assignment	Secondary outcomes: blood lymphocyte count and inflammatory markers
NCT06039527 recruiting	TINO: Loss of Nasal T Cells in Vital and Frail Older Individuals	Healthy young adults, vital older adults, frail elderly	Observational Prospective Cohort	Nasal CD8+ T cells. Secondary: nasal immune cells, cytokines, respiratory tract microbiota
NCT06433037 active not recruiting	Gut-brain Health Effects of PREbiotics in Older Adults with Suspected COgnitive DEcline (PRECODE)	Adults 60-89 years of age with subject cognitive decline and presence of at least 2 risk factors for cognitive decline	Randomized, double-blind, placebo-controlled	Effect on working memory. Secondary: effects on immune parameters (blood inflammatory cytokine panel)
NCT02869048 recruiting	Amyotrophic Lateral Sclerosis and the Innate Immune System	Adult and Older Adult with Amyotrophic Lateral Sclerosis	Prospective Case-Control	Complement activity
NCT01565434 completed	Erythrocyte Complement Receptor 1 and Alzheimer Disease	Adult and Older Adult with a Alzheimer dementia	Observational Prospective Cohort	Functional polymorphism of CR1
NCT01816880 completed	Wellderly Immune Antibodies	Olders 90 years of age and over prior enrolled in the Healthy Elderly Study	Prospective Observational Cohort	Antibodies targeting antigens involved in chronic disease

ID, ClinicalTrials.gov identifier (NCT number).

Debating data have been described about CS and aging (14). Complement C3 and C4 levels have been shown to correlate with age (22). C3/C4 levels reach significantly increased levels in centenarians; moreover, elevated levels of C3 and C4 are significantly associated with metabolic syndrome and abdominal obesity in older adults (22). These data are supported by evidence that C3 is significantly associated with metabolic syndrome and lipid metabolism, obesity and cardiovascular disease (22, 23). Current evidence thus confirm that C3 and C4 have positive relationships with metabolic syndrome and negative relationships with centenarian longevity in the oldest-old (22). Authors documented that, in middle-aged and elderly adults, serum C3 can be considered as a potential biomarker for metabolic dysfunction-associated steatotic liver disease even though C3 levels seem to be not independently associated with liver fibrosis in these patients (24). Healthy centenarians present, thus, with a distinct expression of proteins/pathways reflecting a healthy immune function, with less inflammaging and adequate CS regulation. Notably, recent studies documented that C3 is associated with body composition parameters and sarcopenia in older adults: lower levels of C3 correlated with higher risk of sarcopenia (25). Indeed, C3 is also produced in human myoblasts and high levels of C3 might have a protective effect against inflammatory pathophysiology of muscle loss, mainly in olders (26). Taken together, these findings, arising from both the direct human aging data and the mechanistic conjectures beyond current evidence, connect CS regulatory systems to an age-related immune adaptation suggesting an adaptive recalibration of immune fitness in aging. CS in olders could act like a standby lamp, instantly ready for full illumination whenever needed (27, 28). So, despite immunosenescence, in elderly, the CS remains a frontline defense against pathogens; moreover, CS components are involved in glucose and lipid metabolism, and muscle tissue homeostasis, counteracting aging processes.

### 1.2 CS and neuroinflammation: a double-edged sword

While historically considered to be liver produced, almost all proteins can be synthesized at the central nervous system (CNS) level: CS components can be synthesized locally both constitutively and inducible upon injury and/or infections through the activation pathways (29). Indeed, CS proteins can be enhanced in CNS during the aging process (30, 31). As described, in the developing brain, CS proteins contribute to cognitive ability through synaptic circuit refinement (32). CS, beyond its role in immune response, through proteins like C1q, C3, and C4, regulates essential synaptic pruning during normal brain development (33). Evidence from the literature described the role of C1q in neuroprotection and regulation of inflammation. The long CR1 isoform has been identified as being associated with AD risk: that evidence suggests that AD is associated with insufficient clearance of plaque deposits rather than increased CS-sustained inflammation (20). However, in the CNS, CS might play a role in both tissue healing and repair. The C3 activation fragments serve as inflammatory modulators (C3a) or opsonins (C3b, iC3b, and C3d) and can have pro- and anti-inflammatory functions according to context- and/or receptor-dependent pathways (34). In the context of aging, as documented, chronic and sustained CS activity contributes to the degradation of the blood-brain barrier (BBB), increasing susceptibility to neurodegenerative disorders (35). Published studies document that C3/C3a production increases in the aging brain: the activation of the endothelial C3aR leads to inflammatory pathways by the recruitment of immune cells and increased vascular permeability of BBB (36). The C3a/C3aR signaling thus promotes – at the endothelium level - vascular inflammation and BBB dysfunction while promoting neuroinflammation in aging and neurodegenerative disease (36). Nevertheless, Authors recently documented that constitutive loss of C3 in mice was associated with spontaneous locomotor abnormalities during the aging process: in particular, they described decreased speed and gait ataxia, which can be observed in age-related locomotor issues, without anatomical alterations (32, 37). In addition, in AD models, deficiency of C3 can exacerbate neurodegeneration as well as the accumulation of amyloid beta plaques thus suggesting that C3 is a key player in the clearance of deposits (38). Studies also support the contribution of C3 and C3a to the mechanisms of synaptic remodeling and axon regeneration following nerve injury: in this context, C3aR activation was shown to increase neuronal excitability in large fibers (39). Targeting these pathways could provide key strategies for managing nerve dysfunction and inflammation in peripheral neuropathies like diabetic neuropathy. As well documented, peripheral neuropathies associated with diabetes, along with malignancy and monoclonal gammopathies, are more common in olders than in young people and represent a relevant factor of disability in the elderly: it is thus essential to design potential treatment strategies which could prevent severe disease-damage in difficult-to-treat subjects (40). Evidence from literature described key mechanisms where C5a can exert neuronal regenerative activities: C5a acts as a pro-regenerative pathway on neurons by binding C5aR1 (direct pathway) and promotes the axon outgrowth by inducing from the local fibroblasts the release of crucial neurotrophic factors - Nerve Growth Factor (NGF) and Brain-Derived Neurotrophic Factor (BDNF) - (indirect neurotrophic pathway). However, the C5a-related inflammatory or pro-regenerative outcomes seem to depend on timing, concentration and local milieu (41). In particular, CS fragments (mainly C5a) have a dual role where normal-moderate levels support immune fitness in defense and tissue repair (as pro-regenerative actions), while abnormal and deregulated activation leads to tissue damage by driving chronic inflammation (41). Timing and concentrations thus affect outcomes while, in aged context, the sustained “inflammaging” supports cellular senescence and impaired regenerative signaling (41).

In neurodegenerative diseases such as AD and Amyotrophic lateral sclerosis (ALS), abnormal protein aggregates act as triggers for CS activation and lead to subsequent elevated production of C5a (41). In this context, intriguing findings underscore the C5-C5aR1 axis as a promising target for neurodegenerative diseases: preclinical studies show that C5aR1 antagonists, such as PMX205, mitigate disease progression in AD and ALS animal models by reducing neuroinflammation and preserving synaptic function (42).

According to recent evidence the CS has gained increasing recognition for its highly regulated non-canonical roles in the CNS, particularly in normal CNS development and continuous neuroplasticity in the adult and aging brain (43). It is important to highlight the intracellular dimension of CS activity at CNS level for understanding CS “dual role” in inflammation and repair. According to findings from the literature, CS appears to act within neuronal cells to directly induce their profile rather than just as an external and environmental modulator (44). Notably, the intracellularly active CS has gained increasing attention for its multifaceted pathogenic role in several neurodegenerative and neuroinflammatory diseases affecting elderly populations. Therefore, strategies targeting destructive neuroinflammatory pathways while preserving neuroprotective functions could be crucial for treatment of aging-related neuroinflammatory diseases including AD, Parkinson disease, and Huntington disease, which are all characterized by both the CS-dependent activities, the clearance of aggregated proteins and the inflammatory neurotoxic effects (44).

**Figure 1:**
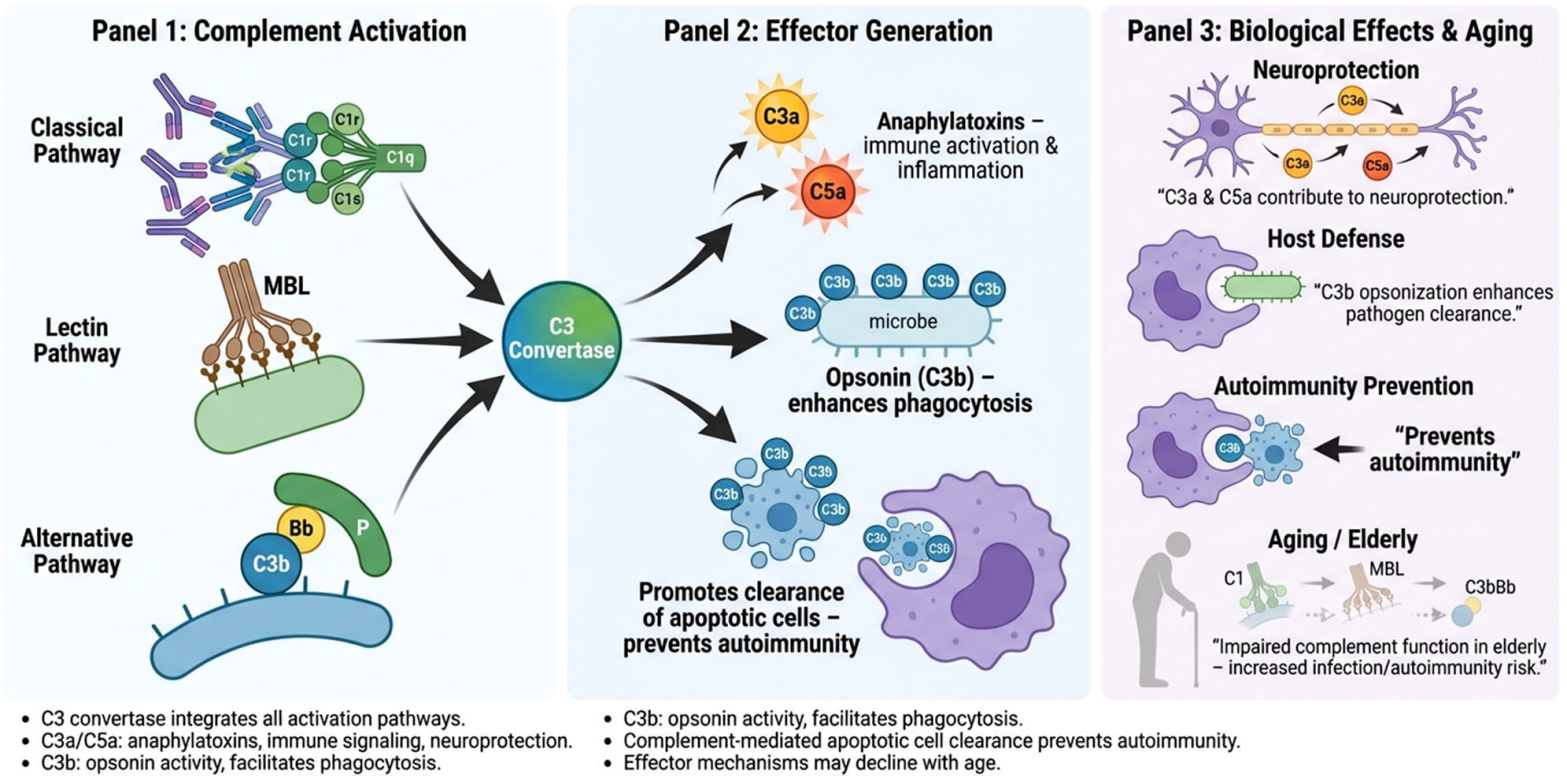
Complement cascade pathways and downstream functions in neuroprotection, host defense, and autoimmunity.

As depicted in **Figure 1**, CS activation pathways and their specific homeostatic biological effects, during aging, could have a functional decline thus disrupting host defense and mechanisms of neuroprotection: in this context, the “aged milieu” goes toward a state of increased risk for infection and autoimmunity. Nevertheless, we support the idea of the “dual role” of CS at nervous system level: a pro-inflammatory role leading to several cellular mechanisms of inflammation and tissue damage, and anti-inflammatory role in neuroplasticity and repair. In this view, evidence on aging-related neuroinflammation suggests that CS remains the main sensor of tissue distress, thus protecting the brain from both infectious pathogens and the accrual of dysfunctional or necrotic biological debris.

### 1.3 CS and autoimmunity in aging: fighting for fitness

Aging-driven immunosenescence is a major risk factor for autoimmune diseases (AIDs) which are mostly prevalent during or after middle age (45). In fact, both the age-associated changes in the self-antigens and the potentially impaired clearance of apoptotic cells lead to the accumulation of aged cell subtypes while promoting susceptibility to AIDs development (45). During aging, several mechanisms could impact AIDs onset and outcome: the age-related comorbidities, the combination therapies, along with environmental factors and genetic abnormalities (12, 46). All these factors strongly influence the risk of AIDs and the overall health outcome in elderly populations. Moreover, comorbidities could negatively affect AIDs-outcome and *vice versa*, supporting a vicious cycle in which immunosenescence and autoimmunity interact in a bidirectional causal pattern (45). Main changes associated with immunosenescence that lead to autoimmunity include autoreactive T cells, Treg dysfunction and abnormal TCR signaling, which is related to thymic involution and impaired thymic output, along with pro-inflammatory and pro-fibrotic activities of senescence-associated secretory phenotype (SASP) of senescent cells. Another crucial hallmark of T cell senescence is the terminally differentiated T cell population without the costimulatory molecule CD28 (47). CD4^+^CD28^-^ T cells have been linked with pro-inflammatory mechanisms and AIDs (48). All these factors lead to chronic low-grade inflammation and increase the predisposition to autoimmunity in the elderly (49). Furthermore, in older subjects, during infections, the accumulation and/or the alterations of self-antigens could increase the risk of cross-reactivity with microbial epitopes - through epitope mimicry - thus complicating the disease outcome and vulnerability (50). However, circulating autoantibodies in elderly are specific for modified antigens and immunoglobulin but, although disease-specific, they could represent just a phenomenon rather than a defined disease (45). Despite frequently showing circulating autoantibodies, centenarians rarely develop clinically defined AIDs (51). In this context, several mechanisms could interact including possible distinct genetic and epigenetic signatures, enhanced regulatory T-cell responses, and IgM autoantibodies with protective functions (52). Moreover, elderly people with AIDs can show a different disease phenotype and treatment-response. For instance, immunosenescence is expected to influence phenotypes and immunological profile of systemic lupus erythematosus (SLE) (53). In late-onset SLE, characteristic inflammatory drivers are attenuated - often due to the downregulation of interferon signaling - resulting in atypical clinical presentation thus complicating the diagnostic process (54). These factors, alongside age-related comorbidities - particularly diabetes and cardiovascular diseases - can lead to inadequate classifications and treatments, suboptimal outcomes, and thus increased cumulative damage (55). Late-onset SLE tends to show a lower disease activity, indolent disease course, and higher disease damage than early-onset SLE (54). However, late-onset SLE in characterized by less frequent hypocomplementemia and more frequent positivity of rheumatoid factor, antiphospholipid autoantibodies, and overlapping anti-SSA positive Sjögren’s syndrome (SS), often complicating with lung disease (56, 57). Evidence recently described that in SS the age of patients appears to be linked with an anti-SSA antigenic specificity characterized by a significant dissociation between a mild systemic activity and a great symptom severity (58). Probably related to the lack of both hypocomplementemia and typical clinical manifestations, elderly patients with SLE tend to have a low disease activity; however, they accrue greater cumulative organ damage. Additionally, in late-onset SLE, survival rates decline with age progression and correlate significantly with the incidence of infections and malignancies. Nevertheless, the normal or mildly reduced complementemia (C3, C4, CH50) in older patients could correlate with less severe renal and hematological manifestations. However, clinical phenotypes and organ involvement in SLE (mainly lupus nephritis) are strongly influenced by the ethnic variations thus showing a rate of occurrence which might persist into older age (57). In late-onset SLE, both anti-SSA positivity and older age at onset appear to independently predict the organ damage accumulation despite the levels of CS components (57). In late-onset SLE, we might hypothesize that the effects of immunosenescence counteract the intensity of the SLE-specific autoimmune response: nevertheless, a typical type I interferon pathway activation could remain prevalent in some patients, leading to classic clinical and laboratory phenotypes which can remain significant in a proportion of patients (57). Proteomic analyses recently showed that complement component 9 (C9) and C1 inhibitor (SERPING1 gene) were to be positively correlated with chronological age in a cohort of healthy men and women (aged up to >90 years) suggesting that expression levels of these proteins regulating immune-response, inflammation, and vascular permeability might be a signature of longevity (59, 60). Moreover, healthy centenarians have been shown to express higher levels of proteins associated with B lymphocyte-mediated immune response and phagocytosis thus supporting the role of a healthy immune system as a differential feature of disease-free centenarians (61). Authors recently documented a relevant CS presence in centenarians’ blood proteome: they identified several proteins belonging to the CS thus suggesting the crucial role of CS in regulating immune responses and promoting resistance to autoimmunity in centenarians (62). These findings are in line with the idea of the dual capability of CS in elderly: promoting inflammatory responses and establishing regulatory conditions thus acting against infections and autoimmunity. It might suggest that a tailored modulation of CS-pathways could represent a crucial mechanism in centenarians’ immune fitness, reducing the risk of AIDs despite the presence of circulating autoantibodies (62). Based on the current knowledge, geroprotective therapies targeting the mechanisms of immunosenescence are just emerging (63). Robust evidence includes fasting, resveratrol, NAD precursors, and other strategies that target aging (64). However, clinical studies need to intensify in terms of effectiveness and safety for humans (63, 64).

## 2 Conclusions

The protective role of CS upregulation in aging remains under investigation. Understanding the potential dual role of CS in aging is crucial to design potential innovative interventions to counteract cellular senescence and inflammation in age-related immune disorders. Novel strategies should preserve the delicate balance of CS activities since unregulated CS inhibition could be certainly detrimental for immune homeostasis in elderly. CS-targeted therapies could be designed for patients with specific CS-driven phenotypes and operate through two distinct mechanisms: targeting destructive inflammatory pathways, which cause disease-damage, and preserving protective functions, which promote both resistance to vulnerability and autoimmunity thus maintaining immune equilibrium in longevity.
